# Gastric metastasis from colorectal cancer: an updated comprehensive review of epidemiology, mechanisms, diagnosis, and management

**DOI:** 10.3389/fonc.2026.1922167

**Published:** 2026-09-11

**Authors:** Chunmei Wang, Chunjing Jie, Yu Su, Zongming Gu, Wenzhong Chen

**Affiliations:** 1 Digestive System Department, Tongren People’s Hospital, Tongren, China; 2 Digestive System Department, Red Flag Hospital Affiliated to Mudanjiang Medical University, Mudanjiang, China

**Keywords:** colorectal cancer, differential diagnosis, gastric metastasis, immunohistochemistry, metastatic tumor, SATB2

## Abstract

Colorectal cancer (CRC) is one of the most common and deadly malignancies worldwide, with metastasis being its most lethal feature. Although the liver and lungs are well-recognized sites of metastasis, gastric involvement as a target organ is extremely rare. The exact incidence remains unclear due to the lack of large-scale epidemiological data, and existing literature consists mainly of case reports and empirical risk estimates. This rarity, combined with the frequent misdiagnosis of gastric metastases as primary gastric cancer in clinical practice, makes gastric metastasis from CRC a unique clinical entity characterized by diagnostic challenges and therapeutic difficulties. Analysis of available case reports indicates that the median age at onset is approximately 65 years, with the left-sided colon being the most common primary site. The interval between diagnosis of the primary tumor and development of gastric metastasis ranges from 6 months to 10 years. Diagnosis primarily relies on a combination of immunohistochemical markers—CK7−/CK20+/CDX2+—while the emerging marker SATB2 further enhances diagnostic specificity. Currently, there is limited high-level evidence-based guidance for treatment; systemic chemotherapy regimens used for metastatic CRC—such as FOLFOX or FOLFIRI combined with targeted therapy—are typically employed. Surgical resection may be considered for isolated, resectable gastric metastases, but overall prognosis remains poor, with a median survival of approximately 12 months. This review comprehensively summarizes the epidemiological characteristics, molecular mechanisms, clinicopathological diagnosis, routes and mechanisms of metastasis, clinical manifestations, diagnostic strategies, differential diagnosis, treatment options, and prognostic factors associated with gastric metastasis from CRC, aiming to provide clinicians with a comprehensive understanding of this rare condition and to serve as a reference for future research and clinical practice.

## 1 Introduction

Colorectal cancer is the third most common cancer worldwide, with approximately 1.9 million new cases and 930,000 deaths annually, making it the second leading cause of cancer-related mortality globally (1, 2). Metastasis is the primary cause of death in CRC patients; approximately 20% are diagnosed with distant metastases at initial presentation, while another 50% of patients with localized disease eventually develop metastatic disease (3). The liver is the most common site of metastasis, affecting around 50% of patients with metastatic CRC, followed by lung involvement in 20-30%. Additionally, the peritoneum, bone, and brain are also frequent sites of metastasis (4). In recent years, significant progress has been made in understanding the molecular mechanisms underlying organotropic metastasis in CRC. Mechanisms such as tumor microenvironment remodeling, epithelial–mesenchymal transition (EMT), metabolic reprogramming, genomic alterations, and immune evasion have been identified as key regulators of tumor cell dissemination to specific organs (3).

However, among various metastatic sites, gastric metastasis from CRC remains extremely rare. A large-scale autopsy study revealed an overall incidence of 0.7%–5.4% for gastric metastases originating from various malignancies, with CRC contributing only a negligible proportion (5). Due to its rarity, gastric metastasis from CRC has long appeared in academic literature solely as isolated case reports, lacking systematic epidemiological studies, standardized diagnostic criteria, and evidence-based treatment guidelines. Reported data indicate that breast cancer and malignant melanoma are the most common primary tumors causing gastric metastases, whereas CRC is not a major source (6). A recent review analyzing gastrointestinal metastatic tumors between 2000 and 2023 found that among 165 patients, the most common primary tumors were breast cancer (50.30%), renal cancer (16.96%), lung cancer (16.36%), and melanoma (12.72%), with CRC failing to rank among the top five (7). This distribution imbalance raises an intriguing scientific question: why does CRC, despite its high incidence, so rarely metastasize to the stomach? Is there a “barrier” effect imposed by the gastric microenvironment on CRC cells, or do CRC cells undergo selective filtration during dissemination? Investigating these questions would not only enhance our understanding of the selective patterns governing organotropic metastasis but also provide a theoretical foundation for developing strategies targeting rare metastatic pathways.

Literature search strategy: We systematically searched PubMed, Web of Science, and CNKI databases for articles published from inception to December 2025, using the following keywords and their combinations: “colorectal cancer,” “colon cancer,” “rectal cancer,” “gastric metastasis,” “stomach metastasis,” “secondary gastric tumor,” and “immunohistochemistry.” We included case reports, case series, and retrospective studies that reported clinicopathological data on colorectal cancer metastasizing to the stomach. Reference lists of included articles were also manually screened to identify additional relevant reports. Based on this strategy, we identified and analyzed over 60 published cases to summarize the epidemiological and clinical features presented in this review.

This article aims to systematically integrate existing knowledge on gastric metastasis from CRC, reviewing the topic from epidemiology to molecular mechanisms, diagnostic approaches to clinical management, and propose future research directions in this field.

## 2 Epidemiological characteristics

### 2.1 Incidence and distribution

The true incidence of CRC metastasis to the stomach is difficult to estimate accurately, primarily due to two factors: first, gastric metastases may be significantly underdiagnosed clinically because they lack specific symptoms; second, large-scale population studies have not treated “gastric metastasis” as an independent analytical variable. Available data mainly come from three sources: autopsy studies, endoscopic screening studies, and clinical case reports. According to indirect estimates from a large-scale autopsy study, the overall incidence of gastric metastases originating from various malignancies ranges from 0.7% to 5.4%, but CRC accounts for only a very small proportion (5). A population-based screening study using upper gastrointestinal endoscopy found that the detection rate of metastatic lesions in the upper digestive tract was approximately 0.026% (approximately 1 in 3,847 examinations), with the stomach being one of the most common sites of metastasis, although only a minority originated from CRC (6). Among metastatic tumors involving the stomach, breast cancer and malignant melanoma are the primary sources, while CRC accounts for less than 5%. Another literature review on gastrointestinal metastases (2000-2023) included 165 patients, with the most common primary tumors being breast cancer, renal cell carcinoma, lung cancer, melanoma, and liver cancer (7).

Based on current literature searches, as of December 2025, there have been over 60 reported cases of CRC metastasizing to the stomach. In 2026, Oh et al. reported a case of synchronous gastric metastasis from sigmoid colon cancer in a 78-year-old male, with the primary tumor located in the sigmoid colon and the gastric metastasis situated on the lesser curvature of the gastric antrum (8). This case suggests that gastric metastasis from CRC can be detected at the time of primary tumor diagnosis. Notably, metastasis of gastric cancer to the colorectum is also rare. A narrative review using the search terms “gastric cancer” and “colorectal metastasis” on PubMed identified only 24 relevant papers and 26 cases of gastric cancer metastasizing to the colorectum (9). This “bidirectional rarity” suggests the existence of some yet incompletely understood selective barriers to cross-regional metastasis among gastrointestinal tumors. A supplementary table summarizing the clinical and epidemiological characteristics of reported cases from the major literature sources, stratified by study type and data source, is provided as **Supplementary Table 1**.

### 2.2 Patient characteristics

Based on the pooled analysis of existing case reports, the clinical characteristics of patients with CRC metastasizing to the stomach exhibit the following patterns.

#### 2.2.1 Age and gender

The median age at onset is approximately 65 years (range 38–86 years), with a slight male predominance (male-to-female ratio of approximately 1.3:1). The age distribution of patients is generally consistent with the overall epidemiological characteristics of primary CRC (6, 7).

#### 2.2.2 Primary tumor site

The left colon (including the sigmoid and descending colon) is the most common primary site, accounting for approximately 60% of all reported cases; rectal involvement follows, accounting for approximately 25%; right-sided colon and transverse colon are less frequently affected. Whether this distribution pattern is related to the anatomical direction of portal venous blood flow remains to be further investigated (8).

#### 2.2.3 Metastatic timing pattern

Gastric metastasis can be classified into two types—synchronous and metachronous. Synchronous metastasis refers to gastric involvement detected at the time of primary CRC diagnosis or within 6 months after diagnosis; metachronous metastasis refers to gastric involvement identified more than 6 months after resection of the primary tumor. Current data indicate that approximately 40% are synchronous and 60% are metachronous. The median interval for metachronous metastasis is 24 months, with a range of 6 months to 10 years (6).

#### 2.2.4 Synchronous metastasis

Approximately 70% of patients have metastases to organs other than the stomach; the liver is the most common synchronous site (approximately 35%), followed by the lungs and peritoneum. Only approximately 30% of patients present with “isolated gastric metastasis,” where the stomach is the sole distant metastatic site (7, 8).

### 2.3 Data limitations

The epidemiological data presented in this review should be interpreted with caution due to several inherent limitations. First, the pooled analysis is based on approximately 60 published cases—a relatively small sample that limits statistical power. The reported percentages (e.g., 60% left-sided colon, 40% synchronous vs. 60% metachronous) should therefore be viewed as approximate trends rather than precise population estimates, particularly given the small sample size and the potential for publication bias. Second, the available data are derived predominantly from case reports and small case series, which are subject to publication bias—cases with unusual presentations or favorable outcomes are more likely to be reported, while unremarkable cases may be underreported. Third, the heterogeneity of data sources poses additional challenges. Autopsy studies (e.g., Menuck & Amberg, 1975) provide historical baseline data but may not reflect current patterns due to advances in diagnostics and treatment. Conversely, contemporary case reports lack standardized data collection protocols. The absence of a centralized international registry further compounds these limitations.

Fourth, the lack of standardized diagnostic criteria across studies introduces heterogeneity in case definitions, IHC marker panels used, and reporting formats, limiting the comparability of findings.

Despite these limitations, given the extreme rarity of this condition, case report-based evidence remains the primary source of clinical knowledge. This review is intended as a narrative review to synthesize existing knowledge and identify knowledge gaps, rather than a formal narrative review or meta-analysis. Establishing an international rare metastasis registry remains a key priority for future research.

## 3 Metastatic mechanisms and organotropism

Why is CRC rarely metastasized to the stomach? The answer to this question may lie in multiple stages of the metastatic process. A study systematically reviewed the molecular mechanisms of organotropic metastasis of CRC, indicating that the metastatic process involves multiple key steps such as tumor microenvironment remodeling, EMT, dissemination of circulating tumor cells (CTCs), and adaptive changes in the target organ microenvironment (3).

### 3.1 Possible routes of metastasis

The theoretical pathways by which CRC cells reach the stomach include the following three:

1. Hematogenous metastasis is the most widely accepted pathway. CRC cells first reach the liver through the portal venous system. If metastatic foci can form in the liver or if the tumor cells successfully evade hepatic clearance, the tumor cells can enter the systemic circulation through the hepatic veins and then reach the stomach wall via the celiac artery and gastric artery. This “roundabout” metastatic path implies that successful gastric metastasis requires the tumor cells to pass through two barriers: the portal–hepatic barrier and the systemic circulation–gastric microenvironment barrier sequentially. This might be one of the reasons why gastric metastasis is much less common than liver metastasis (3, 4).
2. Lymphatic metastasis is another possible pathway. After the regional lymph nodes (such as mesenteric and abdominal lymph nodes) of CRC are involved, tumor cells can retrogradely spread along the lymphatic network to the perigastric lymph nodes and then invade the stomach wall. This pathway can explain some isolated gastric metastasis cases without simultaneous liver metastasis (6).
3. Direct implantation is anatomically feasible, especially for transverse colon cancer. The transverse colon is adjacent to the gastric fundus through the gastrocolic ligament, and the tumor can directly penetrate the serosal layer and invade the stomach wall. However, in clinical cases, gastric metastatic foci are mostly located on the antrum and lesser curvature side of the stomach, rather than the greater curvature side, suggesting that direct implantation is not the main pathway (8).

### 3.2 The “seed–soil” hypothesis from a gastrointestinal perspective

The classic “seed–soil” hypothesis provides an important theoretical framework for understanding organotropic metastasis. Its core idea is that the occurrence of metastasis is not only determined by the intrinsic characteristics of the tumor cells (the “seeds”) but also highly dependent on the suitability of the microenvironment of the target organ (the “soil”) (3). From this perspective, when examining gastric metastasis of CRC, the gastric microenvironment may have certain “unfriendly” characteristics for CRC cells. First, although the gastric lumen is strongly acidic (pH 1.5-3.5), this environment does not directly affect circulating tumor cells (CTCs) during early hematogenous or lymphatic dissemination, as these cells lodge within the submucosal microvasculature or serosa, which maintains a buffered physiological pH. The acidic and protease-rich milieu may only pose a survival challenge to tumor cells during late-stage intraluminal exposure after mucosal ulceration (6). Second, the barrier function of the gastric mucosa—including the gastric mucus layer, tight junctions, and immune cells in the lamina propria (such as macrophages and lymphocytes)—constitutes physical and immunological barriers against CTCs (5). Third, the selective adhesion molecule profile expressed on the endothelial cells of gastric mucosal vessels may be unfavorable for the adhesion and outgrowth of CRC cells. Studies have found that hepatic sinusoidal endothelial cells take up CTCs through the CD36 receptor, forming hepatic micrometastatic foci; it remains unclear whether gastric microvascular endothelial cells express similar uptake molecules (3, 4).

### 3.3 Hypotheses at the molecular level

Although there is currently a lack of direct molecular mechanism studies specifically addressing “why CRC rarely metastasizes to the stomach,” based on the research findings on CRC liver metastasis, the following testable hypotheses can be proposed:

1. Chemokine CXCR4/CXCL12 axis: The CXCR4/CXCL12 signaling axis plays a crucial role in CRC liver metastasis, and the expression level and distribution of CXCL12 in gastric tissue may differ from those in the liver (3).
2. Integrin-mediated organ-specific adhesion: The degree of matching between the integrin subtypes expressed on CRC cells (such as αvβ3, αvβ5, and α2β1) and the corresponding ligands expressed by the vascular endothelium of the target organ determines organ specificity. Collagen I induces EMT by activating the α2β1 integrin–PI3K/AKT pathway, enabling tumor cells to acquire chemotaxis and invasiveness (3). The extracellular matrix composition of gastric microvascular endothelial cells (such as collagen subtype distribution) is significantly different from that of the liver.
3. Metabolic adaptability: CRC cells are characterized by enhanced aerobic glycolysis. The liver, as the first site of metastasis, with its high bile acid and hypoxic metabolic microenvironment, prompts CRC cells to activate the ALDOB enzyme, converting glucose into fructose for metabolism (3). The highly acidic, low-glucose, and hypoxic characteristics of the gastric microenvironment may require metastatic tumor cells to possess distinct metabolic adaptability.
4. Immune surveillance: The lamina propria of the gastric mucosa is rich in macrophages, dendritic cells, and lymphocytes, forming a powerful immune surveillance network. The survival of gastric metastatic tumor cells may depend on effective immune escape mechanisms. Studies have shown that M2-type tumor-associated macrophages account for up to 58% in patients with brain metastasis, forming an immunosuppressive microenvironment by secreting IL-10 and TGF-β (3). Whether similar immune escape mechanisms also play a role in gastric metastasis remains to be clarified.

### 3.4 Key questions remaining to be answered

Based on the above analysis, the following core questions urgently need to be addressed through future research: (1) What is the fate of CTCs in the portal vein–liver pathway—is gastric metastasis necessarily accompanied by liver metastasis? (2) Which molecular markers can distinguish between gastric-dominant and liver-dominant subpopulations of CRC cells? (3) Which factors in the gastric mucosal microenvironment constitute the selective barrier for CRC cells? (4) What is the clonal evolution relationship between gastric metastatic foci and the primary lesion?

## 4 Clinicopathological characteristics and diagnosis

### 4.1 Clinical manifestations

The clinical manifestations of gastric metastasis from CRC are not specific. Most patients are found to have gastric lesions incidentally during examinations for nonspecific digestive tract symptoms. Common symptoms include upper abdominal discomfort or dull pain (approximately 60%), anemia (approximately 35%, mostly due to chronic blood loss), melena or positive fecal occult blood (approximately 25%), nausea and vomiting (approximately 20%), and anorexia and weight loss (approximately 30%). Some patients may have no symptoms at all and only have their gastric lesions discovered accidentally during routine follow-up gastroscopy. Compared with primary gastric cancer, gastric metastasis from CRC is less likely to present with obvious obstructive symptoms, and severe complications such as gastric perforation and acute upper gastrointestinal hemorrhage are also relatively rare (6–8).

### 4.2 Endoscopic characteristics

The morphological manifestations of gastric metastatic tumors under gastroscopy are diverse. The most common type is the protuberant type (60%–70%), which presents as polypoid, lobulated, or nodular protrusions, with a smooth surface or a villous appearance. The second type is the submucosal tumor type (20%–30%), characterized by an inwardly protruding lesion with intact and smooth mucosa, easily misdiagnosed as gastrointestinal stromal tumor (GIST) or leiomyoma (6). The least common types are the flat type and the ulcerative type, with the latter presenting as an ill-defined superficial ulcer. In the cases reported by Oh et al., a 3 cm × 2 cm ulcerative mushroom-shaped mass was visible on the lesser curvature of the gastric antrum under gastroscopy, with a lobulated surface and a central depression and erosion (8). The endoscopic manifestations of this case are representative: gastric metastatic lesions are usually single (approximately 85%), most commonly located in the gastric antrum and the lesser curvature of the gastric body, and most lesions have a diameter of 1–5 cm. The number, size, and location of the metastatic lesions have no clear correlation with the patient’s clinical manifestations and prognosis.

It should be noted that synchronous or metachronous gastric metastasis coexists with gastric cancer. Some scholars have proposed that when a gastric mass is found in patients with confirmed CRC, pathological biopsy and immunohistochemistry should be used to distinguish whether it is gastric metastatic CRC, primary gastric cancer, or both coexisting (6, 9).

### 4.3 Imaging findings

Abdominal contrast-enhanced CT is the most commonly used imaging method for evaluating gastric metastasis. Typical manifestations include focal thickening of the gastric wall or soft tissue masses, which show mild to moderate enhancement after contrast administration, and have a clear boundary from the adjacent gastric wall. However, CT has low sensitivity for small lesions (<1 cm) and is difficult to accurately assess the depth of lesion invasion and its relationship with surrounding structures (7). ^18^F-FDG PET/CT is more sensitive than CT in detecting gastric metastasis, but it also has certain limitations: physiological uptake of normal gastric mucosa may mask small lesions; perigastric inflammatory reactions can lead to false-positive results; and for mucinous adenocarcinoma metastases with low glucose metabolism levels, false-negative results may occur (7). Therefore, PET/CT findings should be combined with endoscopy and pathological examination for comprehensive assessment.

### 4.4 Pathological diagnosis: immunohistochemistry as the gold standard

The pathological diagnosis of gastric metastasis of CRC relies on immunohistochemical (IHC) analysis, which is crucial for differentiating it from primary gastric cancer or other metastatic tumors from other sources.

#### 4.4.1 Morphological features

On routine hematoxylin and eosin (HE) staining, the histological morphology of gastric metastatic CRC is basically the same as that of primary CRC, presenting typical glandular, tubular, papillary, or cribriform structures, with moderately to severely atypical cell nuclei and frequent mitotic figures. An important distinguishing point from primary gastric cancer is that the surrounding mucosa of the metastatic lesion lacks the continuous carcinogenesis process from normal gastric epithelium → intestinal metaplasia → atypical hyperplasia → adenocarcinoma, and the gastric mucosal background usually shows no obvious abnormalities (6).

#### 4.4.2 Immunohistochemical diagnostic criteria

CRC cells specifically express CK20 and CDX2, while CK7 is almost always negative. This combination (CK7−/CK20+/CDX2+) has been widely accepted as a diagnostic criterion for colorectal origin (10, 11). In contrast, primary gastric adenocarcinoma is mostly CK7+, with variable CK20 expression, and CDX2 can be positive in intestinal-type gastric cancer but negative in diffuse-type gastric cancer (10).

#### 4.4.3 Novel marker SATB2

SATB2 is considered a highly specific marker for colorectal origin. Studies have shown that the positive rate of SATB2 in CRC is over 90%, while its expression rate in tumors from other sources is extremely low (10, 12). The introduction of SATB2 in gastric metastasis diagnosis further improves the specificity and sensitivity of the diagnosis (10). Systematic summaries of IHC expression characteristics of different tumor origins have been made, which can help pathologists precisely locate the source.

#### 4.4.4 Other auxiliary markers

Positive villin supports a colorectal origin and has auxiliary diagnostic value for cases with atypical CK20 expression. When CK20 and CDX2 expression are atypical, SATB2 should be a mandatory marker (10).

## 5 Differential diagnosis

When a mass lesion is detected in the stomach of a CRC patient, the process of differential diagnosis is a crucial step in clinical decision-making.

### 5.1 Primary gastric cancer

This is the most important differential diagnosis and the most prone to misdiagnosis. When a gastric mass is detected in a patient with confirmed CRC, it is necessary to first distinguish whether it is primary gastric cancer (possibly a second primary malignancy) or gastric metastatic CRC. The key points for differentiation include: (1) Endoscopic features—metastatic cases are usually single, with clear boundaries, and may present as submucosal tumors; primary cases are often accompanied by multifocal lesions or a background of precancerous lesions; (2) Pathological morphology—the surrounding mucosa of metastatic lesions lacks the continuous evolution process from normal gastric epithelium to dysplasia and then to cancer; (3) Immunohistochemistry—typical primary gastric adenocarcinoma is characterized by CK7+, while colorectal-derived metastatic lesions are CK7-/CK20+/CDX2+/SATB2+ (10, 11). Misdiagnosis may lead to inappropriate surgical scope (total gastrectomy vs. partial gastrectomy) and differences in treatment strategy.

### 5.2 Dual primary cancers

The coexistence of CRC and primary gastric cancer is not extremely rare and should be fully considered in elderly patients. To confirm the presence of dual primary cancers, it is necessary to rule out that one tumor is a metastasis of the other. This usually relies on the morphological comparison of the two tumors by pathology and the analysis of their IHC profile characteristics. If the IHC expression patterns of the two tumors are consistent (for example, both CK7−/CK20+), it supports metastasis rather than dual primary. While discordant IHC profiles (e.g., one CK7−/CK20+ and the other CK7+) suggest the possibility of dual primary malignancies, this rule is not absolute, as phenotypic switching during metastasis can alter CK7 expression. Therefore, in cases of diagnostic uncertainty, advanced molecular profiling—such as next-generation sequencing (NGS) clonality assays, identical KRAS/BRAF mutation matching, or microsatellite status tracking—is required to definitively distinguish true dual primary cancers from a single metastatic origin with clonal evolution. Additionally, molecular typing (such as MSI status) can also provide important clues—if the MSI status of the two lesions is inconsistent, it is more likely to be a case of dual primary (9, 10).

### 5.3 Gastrointestinal stromal tumor

Approximately 20%–30% of CRC metastases to the stomach present as submucosal tumor-like lesions, which are difficult to distinguish from GIST on gastroscopy. Both can present with a spherical or hemispherical appearance that protrudes into the cavity, with a complete or nearly complete mucosa covering the surface. The biopsy forceps have difficulty obtaining sufficient depth of tissue. Endoscopic ultrasound (EUS)-guided puncture biopsy is recommended as the gold standard for differentiation. EUS can clearly show the origin of the lesion from the gastric wall layers (from the submucosa or muscular layer), echo characteristics (GIST is mostly hypoechoic or isoechoic), boundaries, and blood flow distribution. Diagnosis depends on IHC detection of c-KIT (CD117) and DOG1—GIST is almost always positive, while metastatic CRC is negative (6).To overcome the diagnostic challenge of submucosal tumor (SMT)-like metastases, we strongly recommend that endoscopic ultrasound (EUS)-guided fine-needle aspiration (FNA) or deep “biopsy-on-biopsy” sampling techniques be performed when a submucosal lesion is encountered. These approaches provide adequate tissue from the deeper layers of the gastric wall, bypassing the intact overlying mucosa that frequently yields false-negative results on standard superficial pinch biopsies.

### 5.4 Primary gastric lymphoma

It commonly occurs in the antrum and body of the stomach. Under endoscopy, it may present as diffuse infiltration type, ulcerative type, or nodular type. In a few cases, it may show a polypoid appearance. Differentiation from gastric cancer and gastric metastatic cancer mainly relies on pathological biopsy and IHC—lymphoma cells express LCA (CD45) and B-cell or T-cell lineage markers (such as CD20, CD3), while epithelial markers (CK, EMA) are negative (6).

### 5.5 Neuroendocrine tumors

Neuroendocrine tumors that originate in the stomach or metastasize to the stomach can present as submucosal protrusions or polyp-like lesions. Differentiation depends on IHC detection of neuroendocrine markers such as synaptophysin, chromogranin A, and Ki-67, and assessment of biological behavior using a grading system.

### 5.6 Gastric metastatic tumors from other sources

As mentioned earlier, the most common primary malignancies in gastric metastatic tumors are breast cancer, malignant melanoma, lung cancer, and kidney cancer (7). When a gastric mass is clinically detected, a comprehensive inference needs to be made based on the patient’s complete history of the primary cancer. For cases where the primary cancer is unclear, a complete set of IHC markers (including CK7, CK20, CDX2, SATB2, TTF-1, PAX8, mammaglobin, S-100, HMB45, etc.) can assist in locating the primary source (7, 10).

## 6 Treatment strategies

Due to the rarity of CRC metastasis to the stomach, there are currently no specialized diagnostic and treatment guidelines or treatment recommendations based on high-level evidence-based medicine (13). The treatment strategies in clinical practice mainly draw on the overall management framework for metastatic CRC, while fully considering the local specificity of gastric metastasis, and individualized treatment plans are formulated under the multidisciplinary team (MDT) model.

### 6.1 Systemic treatment (basic treatment)

Systemic treatment is the cornerstone therapy for patients with gastric metastasis from CRC. The selection of treatment plan should be based on the guidelines for systemic treatment of metastatic CRC, and the molecular classification of the tumor should also be fully considered (13, 14).

#### 6.1.1 Chemotherapy regimens

FOLFOX (oxaliplatin + fluorouracil + leucovorin) and FOLFIRI (irinotecan + fluorouracil + leucovorin) are the most fundamental standardized chemotherapy regimens for metastatic CRC (mCRC). CAPOX (capecitabine + oxaliplatin) has advantages in outpatient administration. The three-drug combination regimen FOLFOXIRI is suitable for young patients with large tumor burden and good performance status (ECOG 0-1) (14).

#### 6.1.2 Targeted therapy

Stratified selection based on RAS/BRAF gene mutation status:

1. RAS/BRAF wild-type — Anti-EGFR monoclonal antibody (cetuximab or panitumumab) combined with chemotherapy can significantly improve the objective response rate and progression-free survival.
2. RAS or BRAF mutant type — Anti-angiogenic therapy (bevacizumab) combined with chemotherapy.

It is worth noting that in the latest version of the CSCO Colorectal Cancer Diagnosis and Treatment Guidelines (2025 edition), for patients suitable for intensive treatment, a new level-I recommendation of “FOLFOX + encorafenib + anti-EGFR monoclonal antibody” has been added for RAS wild-type and BRAF V600E mutant patients. For patients who cannot tolerate intensive treatment, a reduced-dose regimen of encorafenib + anti-EGFR monoclonal antibody can be selected (13).

#### 6.1.3 Immunotherapy

For patients with microsatellite instability-high (MSI-H) or deficient mismatch repair (dMMR), immune checkpoint inhibitors are the preferred treatment. In the 2025 CSCO guidelines update, for mCRC patients with MSI-H/dMMR, the first-line treatment was adjusted from level I recommendation to “nivolumab ± ipilimumab” (13). For second-line treatment, a level III recommendation of fluorouracil–tipiracil + bevacizumab was added to the second-line treatment options in the palliative care group.

It is worth noting that the STELLAR-303 study presented at the 2025 ESMO conference showed that for refractory patients with microsatellite stable (MSS) mCRC, zanzalintinib (a multi-target TKI) combined with atezolizumab had statistically significant improvements in PFS (3.7 months vs. 2.0 months) and OS (10.9 months vs. 9.4 months, HR = 0.80) compared with standard treatment with regorafenib (15).

### 6.2 Local treatment

There is considerable controversy over whether surgical resection should be performed for gastric metastases. Due to the lack of prospective randomized controlled studies, current clinical decisions are mainly based on the accumulation of case reports and analogical reasoning regarding the treatment strategies for liver/lung oligometastases (6, 7).

Potential indications for surgical resection:

1. isolated gastric metastasis with no other unresectable liver/lung metastases;
2. the primary tumor has undergone R0 resection or can be achieved simultaneously with R0 resection;
3. the patient has a good performance status (ECOG 0-1) and can tolerate surgery;
4. the gastric metastasis causes obstruction, bleeding, or perforation and other complications;
5. the prediction of the patient’s response to systemic treatment is not ideal (7, 8).

Surgical methods: Gastric wedge resection or partial gastrectomy (distal or proximal gastric resection) are the most commonly used procedures. Total gastrectomy is only applicable to patients with multiple gastric metastases or when the location of the metastasis is not conducive to partial gastrectomy (8). For example, in the synchronous case reported by Oh et al., the patient underwent radical distal gastrectomy and anterior resection of the sigmoid colon, with postoperative IHC confirming the gastric lesion as metastatic from colorectal origin (8).

### 6.3 MDT treatment strategy

Given that the treatment of CRC with gastric metastasis involves multidisciplinary collaboration among oncology, gastrointestinal surgery, radiotherapy, imaging, and pathology departments, it is recommended to develop individualized treatment strategies within the framework of MDT (13, 14). MDT decisions should comprehensively consider the following factors: the patient’s overall condition (ECOG score, nutritional status, comorbidities), molecular classification (RAS/BRAF/MSI status), tumor burden (number and size of gastric metastases, synchronous metastasis), previous treatment response, and the patient’s treatment preference.

The recommended treatment decision-making process is as follows: first, conduct RAS/BRAF/MSI molecular testing and comprehensive imaging staging → if there is MSI-H/dMMR, prioritize immunotherapy → if MSS/pMMR and RAS/BRAF wild-type, consider anti-EGFR monoclonal antibody combined with chemotherapy → if RAS/BRAF mutant, consider bevacizumab combined with chemotherapy → on the basis of systemic treatment, assess the resectability of gastric metastases → for patients suitable for surgery, perform local gastric resection after effective control of systemic lesions by systemic treatment (13, 14).

## 7 Prognostic analysis

The prognosis of CRC with gastric metastasis is generally poor, but the available data mainly come from case reports, and the statistical power is limited. Based on the current literature, the following trends can be summarized:

### 7.1 Survival data

The median overall survival time for patients with CRC with gastric metastasis is approximately 12 months, which is comparable to the overall prognosis of gastric metastatic tumors from other primaries (usually ≤1 year), indicating statistical parity rather than a distinct survival advantage (6, 7).

### 7.2 Prognostic factors

1. R0 resection is the most definitive favorable prognostic factor to date (8).
2. Concurrent metastasis status — patients with isolated gastric metastasis (without distant metastasis to the liver or lungs, etc.) have a significantly better prognosis than those with multiple distant metastases (7).
3. Molecular typing — patients with MSI-H/dMMR and RAS/BRAF wild-type have significantly longer survival periods than those with MSS/pMMR or RAS/BRAF mutant types (13).
4. Timing of metastasis — metachronous metastasis has a better prognosis than synchronous metastasis, which may be related to the fact that metachronous metastasis represents the “delayed” form of recurrence after the primary tumor is removed and has relatively indolent biological behavior (6).
5. Performance status (ECOG score) and nutritional status are also important prognostic factors.

It is worth noting that, compared with gastric metastatic CRC, the overall prognosis of patients with colorectal metastasis from gastric cancer is worse. A series study involving 14 cases showed that the median OS of patients with colorectal metastasis from gastric cancer was 11 months, and the 2-year OS was 18.5%. This difference may be related to the more aggressive biological behavior of gastric cancer (9, 12).

## 8 Future outlook

### 8.1 Multimodal omics research to elucidate the molecular mechanisms of gastric metastasis

By utilizing single-cell sequencing, spatial transcriptomics, and proteomics techniques, a systematic analysis should be conducted to decipher the clonal evolution relationship between CRC gastric metastases and primary lesions, as well as the molecular determinants of tumor microenvironment characteristics and organotropic tendencies (3). By comparing the multimodal data of paired primary lesions, liver metastases, and gastric metastases, it is expected to identify the key molecules driving gastric-specific metastasis.

### 8.2 Molecular basis of the “barrier effect” in the gastric microenvironment

From the perspective of the host microenvironment, a systematic study should investigate which factors in the gastric mucosal microenvironment have a barrier effect on CRC cells, and how metastatic CRC cells overcome these barriers (3, 5). These findings would not only contribute to understanding the mechanism of gastric metastasis in CRC but may also provide new ideas for developing strategies to prevent other organ metastases.

### 8.3 Application of liquid biopsy in the monitoring of rare metastases

Circulating tumor DNA (ctDNA) technology offers non-invasive methods for early detection of rare metastases and monitoring of treatment responses. Through regular ctDNA monitoring, gastric metastases may be detected before imaging evidence appears, thereby creating opportunities for early intervention. Additionally, ctDNA can be used to track the dynamic evolution of tumor subclones (13).

### 8.4 Optimization of therapeutic strategies

With the continuous enrichment of systemic treatment options for mCRC (BRAF inhibitors, HER2-targeted therapy, PD-1/PD-L1 inhibitors, TKIs, etc., with new drugs continuously emerging), from a translational perspective, we advocate that gastric metastatic lesions in CRC patients should not be routinely treated as mere extensions of systemic disease. In our view, the stomach as a metastatic sanctuary site warrants dedicated investigation for several reasons: First, the distinct immune composition of gastric mucosa—rich in IgA-producing plasma cells and regulatory T cells—may modulate responses to immunotherapy in ways that differ from other metastatic sites; second, the relative accessibility of the stomach via endoscopy offers a practical advantage for serial biopsy and real-time monitoring of clonal evolution, which is logistically more challenging for liver or lung lesions. Therefore, we propose that patients with isolated gastric metastasis should be considered a distinct subgroup in future clinical trials, with separate stratification and analysis, rather than being pooled with other mCRC patients. The optimal systemic treatment strategy for rare anatomical site metastases still requires further evaluation. Prospective cohort studies or multicenter retrospective studies should be conducted to compare the efficacy differences of different treatment strategies in patients with CRC gastric metastasis (13, 14).

## 9 Conclusion

Gastric metastasis from CRC is a rare clinical form of distant metastasis that is challenging to diagnose and has limited treatment options. Although only approximately 60 cases have been reported so far, it holds significant diagnostic value in clinical practice—misdiagnosing it as primary gastric cancer may lead to unnecessary total gastrectomy, thereby missing the best opportunity for systemic treatment. This article comprehensively integrates the epidemiology, metastasis mechanisms, diagnosis, differential diagnosis, treatment, and prognosis knowledge in this field, and proposes a hypothesis framework regarding the potential “barrier effect” of the gastric microenvironment on CRC cells and its molecular basis. The main conclusions are as follows:

1. Immunohistochemical markers CK7−/CK20+/CDX2+ combined with SATB2 are the gold standard for confirming colorectal origin;
2. Treatment should be based on the systemic treatment plan for metastatic CRC, and surgical intervention can be considered for isolated gastric metastasis patients after strict screening;
3. The prognosis is generally poor, and R0 resection and favorable molecular typing (MSI-H, RAS/BRAF wild-type) are favorable prognostic factors.

Looking to the future, the most urgent work in this field is to establish a multicenter rare metastasis registration system, promote multi-omics research to clarify the molecular mechanisms of gastric tropism, and on this basis, formulate individualized precision treatment strategies. Although the “encounter” between CRC and the stomach is extremely rare, it is precisely this rarity that contains scientific issues, providing a unique window for us to understand the selective rules of tumor organ-directed metastasis and develop innovative intervention strategies. We believe that the lessons learned from studying this rare metastatic pathway—particularly the concept of microenvironmental resistance as an active selective force—may ultimately inform broader principles of metastatic biology and refine our approach to oligometastatic disease across multiple tumor types.
